# Atypical Imaging Findings of Nonketotic Hyperglycemic Hemichorea: A Case Report and Review of the Literature

**DOI:** 10.7759/cureus.34269

**Published:** 2023-01-27

**Authors:** Christopher M Stevens, Kevin Malone, Arielle Degueure, Amro Saad Aldine, Octavio Arevalo

**Affiliations:** 1 Interventional Radiology, Louisiana State University Health Sciences Center, Shreveport, USA; 2 Biomedical Engineering, Louisiana State University Health Sciences Center, Shreveport, USA; 3 Medicine, Louisiana State University Health Sciences Center, Shreveport, USA; 4 Neuroradiology, Louisiana State University Health Sciences Center, Shreveport, USA

**Keywords:** basal ganglia, nonketotic hyperglycemic hemichorea, magnetic resonance imaging, computed tomography, imaging, ganglia, basal, hemichorea, hyperglycemic, nonketotic

## Abstract

Nonketotic hyperglycemic hemichorea (NH-HC) is a rare condition presenting in the clinical setting. Brain imaging plays an important role in diagnosing NH-HC, which typically shows basal ganglia changes contralateral to the side of the hemiballismus/hemichorea. Only a few articles in the literature have reported normal pertinent magnetic resonance/CT findings in patients presenting with NH-HC. To the authors' knowledge, no cases in the literature have reported basal ganglia changes solely observed on CT but not on MRI in patients presenting with NH-HC. Herein, we describe a unique case in which a CT of a patient presenting with NH-HC demonstrated basal ganglia abnormalities with negative MRI findings.

## Introduction

Nonketotic hyperglycemic hemichorea (NH-HC) is a rare metabolic syndrome with a prevalence of less than one in 100,000 people [1]. NH-HC is characterized by a triad of nonketotic hyperglycemia, basal ganglia hyperintensity on MRI or high density on CT, and unilateral involuntary jerky (choreiform) movements affecting the upper and lower limbs contralateral to the side of the lesion [2,3]. NH-HC was first described by Bedwell in 1960 and presents typically in older diabetic patients [4,5]. Risk factors include uncontrolled blood glucose, female gender, advanced age, and Asian descent [6,7]. A 2015 meta-analysis reported that the average age of patients who presented with NH-HC was 71 years and the diagnosis female-to-male ratio was 1.8:1 [7].

An important means of diagnosing NH-HC is brain imaging with MRI or CT. Common imaging findings of NH-HC patients include the presence of unilateral basal ganglia abnormalities contralateral to the patient's chorea movements [8,9]. A study conducted in 2020 reported that MRI was roughly 16.5% more sensitive in detecting NH-NC than CT, as MRI readings were consistent with findings of basal ganglia lesions in 95.33% of NH-HC patients, while CT reported a positive finding in 78.86% of these patients [9]. Along with this study, less than five articles in the literature have reported negative CT and MRI imaging findings of a basal ganglia lesion in patients with NH-HC [10-12]. Herein, we describe a unique case in which a CT of a patient presenting with NH-HC demonstrated basal ganglia abnormalities with negative MRI findings. To our knowledge, there are no case reports in the literature that have reported a positive finding of a basal ganglia lesion on CT but a negative finding on MRI in patients presenting with NH-HC.

## Case presentation

A 75-year-old African American female with a past medical history of depression, hypertension, and diabetes presented to the emergency department with a chief complaint of involuntary movements of her right arm and leg for two days duration. The patient was initially evaluated at an outside facility and the workup for a stroke was negative at the time.

The patient denied fevers, chills, recent travel, family history, over-the-counter drug use, or changes in speech. On arrival, the patient’s blood pressure was 182/11 mmHg, with a heart rate of 102 bpm, and a blood glucose level of 353 mg/dL. Large amplitude involuntary movements of the right upper extremity were noted on the exam with maintained sensation and strength in all extremities.

**Figure 1:**
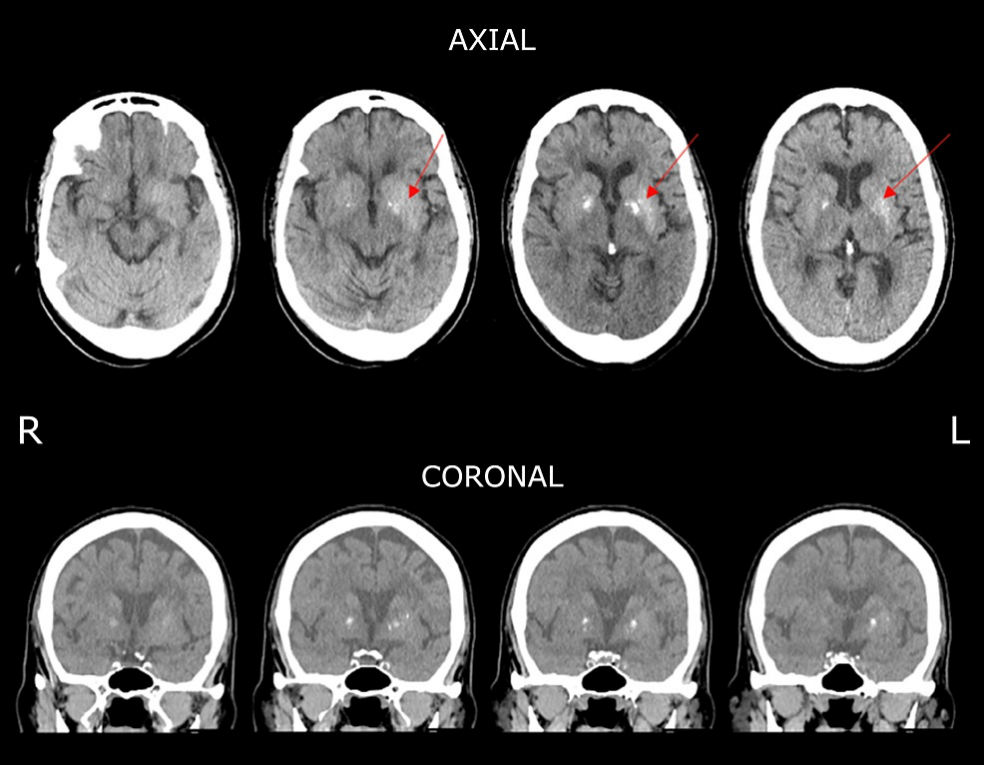
Noncontrast CT of the head showing bilateral basal ganglia calcification with hyperattenuation of the left putamen (red arrows)

A stroke code was promptly called and an emergent CT angiography (CTA) of the head and neck was obtained, which showed no evidence of vessel occlusion or stenosis. The non-contrast CT showed bilateral basal ganglia calcification with hyperattenuation of the left putamen and evidence of age-related parenchymal volume loss and chronic microvascular ischemia (Figure 1). No acute intracranial hemorrhage or large arterial territory infarction was seen (Video 1).

**Video 1:** CT media

**Figure 2:**
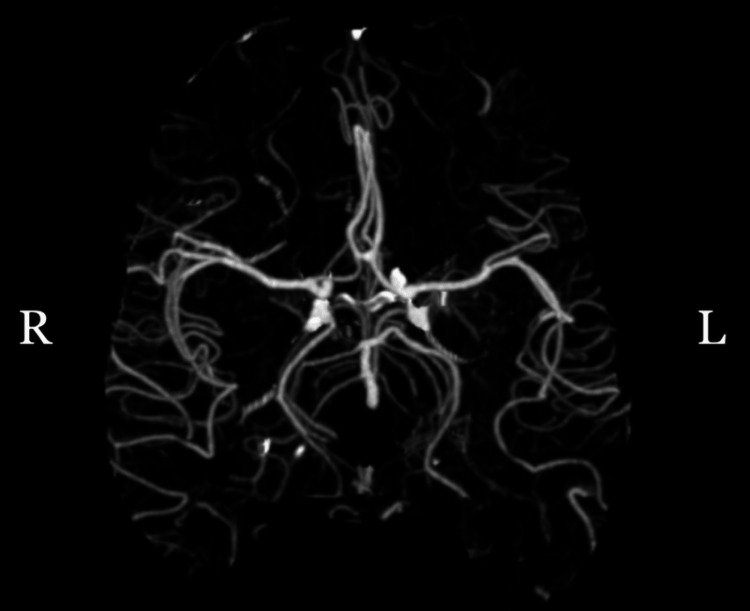
No vascular abnormality is seen on the brain MRI

The patient was treated with five units IV and 1 L Normosol®-R to manage initial glucose levels and was given 10 mg of olanzapine, which promptly managed the patient's hemiballismus. The patient was strictly monitored for glucose control and placed on a sliding insulin scale where blood glucose was maintained under 250 mg/dL. The MRI obtained six hours later and prior to the adequate normalization of the blood glucose level or the resolution of the symptoms failed to correlate with the CT findings and did not show left putamen signal intensity changes on T1/T2 or diffusion-weighted images (Figure 2 and Video 2).

**Video 2:** MRI media

Due to the presence of the hyperattenuation of the left putamen on CT with no evidence of large vessel occlusion/hemorrhage, and the patient's elevated blood glucose levels with chorea-hemiballismus movements, the patient was given the diagnosis of NH-HC. After being evaluated by the medicine and neurology services, the patient was discharged to a rehab facility on metformin and insulin to control her blood glucose levels.

## Discussion

NH-HC is a rare metabolic syndrome typically characterized by a triad of nonketotic hyperglycemia, the presence of basal ganglia hyperintensity on MRI or high density on CT, and unilateral involuntary jerky movements affecting the upper and lower limbs contralateral to the side of the basal ganglia abnormalities [1-3].

While NH-HC is seen during episodes of nonketotic hyperglycemia, the exact pathology is unknown [13]. There are several possible proposed mechanisms of NH-HC, including disruption of the blood-brain barrier, petechial hemorrhage, influence of the post-menopausal state, reduced gamma-aminobutyric acid (GABA), and mineral deposition [9,14].

Hyperglycemia and hyperviscosity have been proposed to disrupt the blood-brain barrier causing intracellular acidosis and regional metabolic failure. Vasculopathy was hypothesized to be a possible mechanism due to the hyperviscosity of blood, which leads to latent ischemia of the striatum and subsequent dyskinesia [15]. Although this hypothesis was not confirmed in cases with unilateral lesions, it was partially supported by a striatal biopsy after hyperglycemia-related hemichorea, which showed neuron loss, gliosis, and reactive astrocytosis [16].

Petechial hemorrhages have been described as another possible explanation for this disorder with several cases having shown an association with striatal infarction [16-18]. One such case describes the post-mortem examination of putaminal tissue revealing petechial hemorrhage in a case of NH-HC [17].

Increased sensitivity of the post-menopausal dopamine receptor can possibly trigger hyperkinesis in an appropriate setting [19]. Such a mechanism has been demonstrated in diabetic rats, showing that dopaminergic neuron metabolic activity may decrease, causing dopaminergic receptors' hypersensitivities in hyperglycemic conditions [20]. Post-menopausal gonadal hormone withdrawal, resulting in a reduction of brain dopamine receptors, may produce an imbalance of GABA type A receptors in the output pathways of the striatum, furthering this hyperglycemic effect [21,22]. This may help to explain why NH-HC is more commonly seen in post-menopausal women.

There is decreased GABA in the thalami/striatum due to the non-availability of acetoacetate for GABA conversion in the nonketotic state. GABA is used as an alternative energy substrate during a hyperglycemic crisis and its depletion leads to thalamic disinhibition and hyperkinesia [15,23]. One case report provided a possible explanation for delayed NH-HC in a 70-year-old man after hyperglycemia correction [24]. While not in the ketotic state, ketone bodies may still play a role in hyperglycemia-related hemichorea. The authors postulated that the absence of insulin leads to hyperglycemia and then the release of free fatty acids from the adipose tissue, which are then metabolized to acetoacetate and β-hydroxybutyrate (ketone bodies). Acetoacetate can be used as a GABA substitute to compensate for the hyperglycemia-induced GABA depletion temporarily.

Mineral deposition, such as calcium or other depositions, has also been proposed as a potential causative factor. Nath et al. demonstrated calcification and mineralization of neurons on pathological examination of a patient diagnosed with hemichorea-hemiballism (HCHB) [25]. However, this was in association with focal microhemorrhages, suggesting petechial hemorrhage. As mentioned earlier in this article, petechial hemorrhage is another possible hypothesis.

It is still unclear what the specific underlying causative factor is. Most reports seemingly present cases with a combination of the various explanations discussed above. It may be as likely that several underlying pathologic causes may be culprits in causing this mechanism, each resulting in a similar HCHB but possibly causing a varied presentation on imaging; however, this is difficult to ascertain.

Quickly decreasing glucose levels with insulin and fluids, along with anti-chorea treatment, has shown to be the best treatment method for NH-HC so far. A recent study reported that approximately 26% of NH-HC patients can achieve a resolution of symptoms by controlling their blood glucose alone but adding in an anti-chorea treatment with the glucose treatment increased the outcome to 76% of patients [9]. Common anti-chorea treatment drugs used in conjunction with glucose treatment include haloperidol, clonazepam, tetrabenazine, and tiapride, with haloperidol being the most frequent [9]. The timeframe for the resolution of symptoms varies. Some patients have shown to experience a resolution of symptoms within days after starting treatment while others do not show any signs of progress until months after starting medication [26,27]. In the case presented, the patient did not have an immediate resolution of symptoms after being administered metformin and insulin.

As previously stated, MRI and CT are the typical imaging modalities used to assist in diagnosing NH-HC. MRI is the preferred imaging, which typically demonstrates signal changes, particularly in the putamen or the caudate [28,29]. MRI T1 hyperintensity is the most consistent finding in NH-HC. Myelin destruction would also present with a high T1 signal; however, myelin destruction has not been seen on pathology reports in previous case reports for NH-HC. A much more likely explanation would be reactive astrocytosis and an abundance of gemistocytes causing the high T1 signal on the MRI; this would be further supported by the reversibility of this disorder on imaging [30]. Other suggested causative factors for this particular T1 hyperintensity include immobilized water molecules, flow-related enhancement, and paramagnetic substances in hemorrhagic tissue, free radicals, lipids, or melanin [31]. For CT, hyperdensity in the striatal region is the most common finding [13,32]. Although MRI is more sensitive and has been shown to perform better at producing positive imaging findings in NH-HC patients, CT is still performed in patients presenting with NH-HC-like symptoms since there is a possibility that CT and MRI may detect abnormalities in different regions of the basal ganglia [9].

NH-HC patients who lack imaging abnormalities in the basal ganglia on both CT and MRI imaging are sporadic and have only been reported less than five times in the literature [10-12]. Chang et al. suggested that the lack of imaging may result from a new subtype classification of NH-HC [10]. The fact that the MRI findings did not match the CT findings in our patient is unique. This is abnormal since MRI has been shown to be more sensitive than CT in having positive image results for a basal ganglia lesion in patients with NH-HC [9]. Therefore, it is difficult to positively state why such a presentation may have occurred. This presentation may have resulted from a rare subtype or non-classical presentation similar to the suggestion of Chang et al. [10]. One suggestion may be reversibility between the time when the CT was taken and the moment when the MRI was obtained. This, however, is unlikely due to the brevity of time between the CT and the MRI and the persistence of symptoms, suggesting another pathology is at work. Hemorrhages and calcifications are more readily detected on CT than in MRI and are more distinguished. This idea, combined with the fact that this may possibly be a non-classical pathology of NH-HC, is more likely the causative factor for a noncontributory MRI, as seen with this patient.

## Conclusions

The case report as described, to the authors' knowledge, has not been seen in the literature: NH-HC patient who showed a positive basal ganglia lesion on CT but not on MRI imaging. This is abnormal since MRI has shown to be more sensitive than CT in having positive image results for a basal ganglia lesion in patients with NH-HC. Therefore, it is difficult to positively state why such a presentation may have occurred. This may have been a result of a rare subtype or non-classical presentation. In addition to the abnormal NH-HC presentation, this report serves as a literature review of the current hypotheses that discuss possible explanations behind the pathophysiology of NH-HC, which is still not clearly understood. More future investigations need to be done to figure out the exact mechanism of NH-HC.
